# The Impact of Entropy and Solution Density on Selected SAT Heuristics

**DOI:** 10.3390/e20090713

**Published:** 2018-09-17

**Authors:** Dor Cohen, Ofer Strichman

**Affiliations:** Information Systems Engineering, IE, Technion, Haifa 3200003, Israel

**Keywords:** SAT, entropy, solution-density

## Abstract

We present a new characterization of propositional formulas called *entropy*, which approximates the freedom we have in assigning the variables. Like several other such measures (e.g., back-door and back-door-key variables), it is computationally expensive to compute. Nevertheless, for small and medium-size satisfiable formulas, it enables us to study the effect of this freedom on the impact of various SAT heuristics, following up on a recent study by C. Oh (Oh, SAT’15, *LNCS 9340*, 307–323). Oh’s findings were that the expected success of various heuristics depends on whether the input formula is satisfiable or not. With entropy, and also with the measure of *solution density*, we are able to refine these findings for the case of satisfiable formulas. Specifically, we found empirically that satisfiable formulas with small entropy “behave” similarly to unsatisfiable formulas.

## 1. Introduction

In [1], Oh examined the impact of various key heuristics in competitive SAT solvers (i.e., solvers of the propositional satisfiability problem). His key findings were that the average success of those heuristics depends on whether the input formula is satisfiable or not. In particular the effect of the *deletion strategy*, *restart policy*, *decay factor*, and *database reduction* is different, on average, between satisfiable and unsatisfiable formulas. This observation can be used for designing solvers that specialize in one of them, and for designing a hybrid solver that alternates between SAT/UNSAT “modes”. Indeed certain variants of COMiniSatPS [1] work this way.

We do not see an a priori reason to believe that the SAT/UNSAT divide—corresponding to the distinction between zero or more solutions—explains best the differences in the effect of the various heuristics (we note that while proving Unsat and Sat belong to separate complexity classes, there is no known connection of this fact to effectiveness of heuristics). In this work we investigate further his findings, and show empirically that there are more refined measures (i.e., properties of the input formula) than the satisfiability of the formula, that predict better the effectiveness of these heuristics. In particular, we checked how it correlates with two measures of satisfiable formulas: the *entropy* of the formula (to be defined below), which approximates the freedom we have in assigning the variables, and the *solution density* (henceforth *density*), which is the number of solutions divided by the search space. Our experiments show that both are correlated with the effectiveness of the heuristics, but the entropy measure seems to be more consistent: *in all experiments, with both random and industrial-like benchmarks, heuristics that are better for unsat formulas are also better for formulas with low entropy*. The results for density are less consistent.

Like several other measures that were proposed in the past (e.g., back-door variables and back-door key variables [2]), both measures are hard to compute. They require solving a #p problem over a SAT formula, and hence at least for now we cannot see a direct application of these results for faster SAT solving of industrial formulas. This does not mean that these results are useless in the long run, however. In particular our contributions are:

1. Suggesting the concept of entropy as a new proxy to the freedom of variables,
2. Showing evidence that for satisfiable formulas there are better predictors of the effectiveness of various SAT heuristics than the sat/unsat dichotomy suggested by Oh, and in particular that *entropy* predicts hardness consistently across all those heuristics (albeit not in all cases with strong statistical significance), and
3. Setting the foundations for future research into approximating entropy fast, which may eventually indeed lead to constructing faster portfolios based on entropy-based hardness prediction.

We continue in the next section by describing the concept of entropy and its relevance to SAT formulas. In Section 3 we explain the statistical methods that we used in this research. Section 4 is the main section of this article. It describes in detail our experimental setting and our empirical findings. Our conclusions from this research appear in Section 5.

## 2. Entropy

Let φ be a propositional CNF formula, var(φ) its set of variables and lit(φ) its set of literals. In the following we will use $v,\bar{v}$ to denote the literals corresponding to a variable *v* when the distinction between variables and literals is clear from the context. If φ is satisfiable, we denote by r(l), for l∈lit(φ), the ratio of solutions to φ that satisfy *l*. Hence for all v∈var(φ), it holds that $r\left(v\right)+r\left(\bar{v}\right)=1$. We now define:

**Definition** **1** (variable entropy). *For a satisfiable formula φ, the* entropy *of a variable v∈var(φ) is defined by*

(1) $$e\left(v\right)≐-r\left(v\right)\log_{2}r\left(v\right)-r\left(\bar{v}\right)\log_{2}r\left(\bar{v}\right)\;.$$

*where $0\cdot \log_{2}0$ is taken as being equal to 0.*

**Figure 1:**
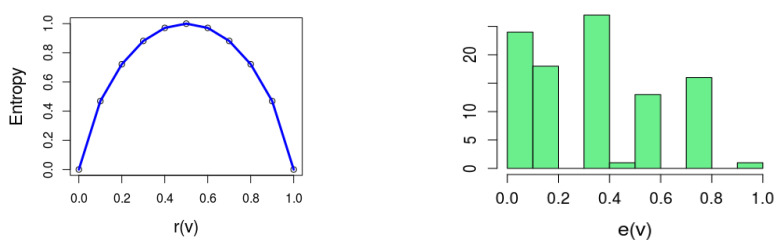
(**Left**) Depicting the entropy function (1); (**Right**) The distribution of e(v) of a formula with 100 variables.

This definition is inspired by Shannon’s definition of entropy in the context of *information theory* [3]. Figure 1 (left) depicts (1).

Intuitively, entropy reflects how “balanced” a variable is with respect to the solution space of the formula. In particular e(v)=0 when r(v)=0 or r(v)=1, which means that $\varphi \Rightarrow \bar{v}$ or φ⇒v, respectively. In other words, e(v)=0 implies that *v* is a *backbone* variable, since its value is implied by the formula. The other extreme is e(v)=1; this happens when $r\left(v\right)=r\left(\bar{v}\right)=0.5$, which means that *v* and $\bar{v}$ appear an equal number of times in the solution space.

**Definition** **2** (formula entropy)**.** *The entropy of a satisfiable formula is the average entropy of its variables.*

As an example, Figure 1 (right) is a histogram of e(v) for a particular formula φ, where for 24 out of the 100 variables r(v)=0.

**Entropy is hard to compute**: Let #SAT(φ) denote the number of solutions a formula φ has. Then it is easy to see that

(2) $$r\left(v\right)=\frac{\#(\varphi \wedge v)}{\#\varphi }\;\mathrm{and}\;r\left(\bar{v}\right)=1-r\left(v\right)\;.$$

Hence computing e(v) amounts to two calls to a model counter. However, since the denominator #φ is fixed for φ, computing e(φ) amounts to |var(φ)|+1 calls to a model counter. Since model counting is a #P problem, we can only compute this value for relatively small formulas.

## 3. A Preliminary: Standardized Linear Regression

We assume the reader is somewhat familiar with linear regression. It is a standard technique for building a linear model $\hat{y}={\hat{\beta }}_{0}+{\hat{\beta }}_{1}x$, where $\hat{y}$ in our case is a predictor of the number of conflicts, and *x* is either the entropy or the density of the formula. We will focus on two results of linear regression: the value of ${\hat{\beta }}_{1}$ and the *p*-value. The latter is computed with respect to a *null hypothesis*, denoted $H_{0}$, that ${\hat{\beta }}_{1}=0$, and an alternative hypothesis $H_{1}$. $H_{1}$ can be either the complement of $H_{0}$ (${\hat{\beta }}_{1}\neq 0$) or a “one-sided hypothesis”, e.g., $H_{1}:{\hat{\beta }}_{1}>0$. In the former case, $p=2Pr(Z\leq z|H_{0})$, where Z∼N(0,1) and $z=\frac{{\hat{\beta }}_{1}-0}{\mathrm{std}({\hat{\beta }}_{1})}$. The ‘0’ in the numerator comes from the specific value in $H_{0}$. In other words, assuming $H_{0}$ is correct, the *p*-value indicates the probability that a random value from a standard normal distribution N(0,1), is less than *z*, the standardized value of ${\hat{\beta }}_{1}$. In the latter case $p=Pr(Z\leq z|H_{0})$.

We list below several important points about the analysis that we applied.

- **Standardization** of the data: given data points $X≐x_{1},\ldots ,x_{n}$, their standardization $X^{\prime }≐x_{1}^{\prime },\ldots ,x_{n}^{\prime }$ is defined for 1≤i≤n by
  $$x_{i}^{\prime }=\frac{x_{i}-\bar{x}}{\sigma }\;,$$
  where $\bar{x}$ is the average value of *X* and σ is its standard deviation. Now $X^{\prime }$ has no units, and hence two standardized sets of data are comparable even if they originated from different types of measures (in our case, entropy and density). All the data in our experiments was standardized.
- **Bootstrapping**: Bootstrapping, parameterized by a value *k*, is a well-known technique for improving the precision of various statistics, such as the confidence interval. Technically, bootstrap is applied as follows: Given the original *n* samples, uniformly sample it *n* times with replacement (i.e., without taking the sampled points out, which implies that the same point can be selected more than once); repeat this process *k* times. Hence we now have n·k data points. For our experiments we took k=1000, which is a rather standard value when using this technique. In each of the experiments that will be reported later on n=5000, hence we have a total of $5\times 10^{6}$ data points for each experiment.
- **Two regression tests**: The entropy and density data consists of pairs of the form 〈entropy,conflicts[i]〉, and 〈density,conflicts[i]〉, respectively, where i∈{1,2} is the index of the heuristic (e.g., in Section 4.3 we will compare the effectiveness of two restart strategies, so the indices 1 and 2 refer to those strategies). Hence the corresponding data is four series of points $\left(e_{1},c_{1}\left[i\right]\right),\ldots ,\left(e_{n},c_{n}\left[i\right]\right)$, and $\left(d_{1},c_{1}\left[i\right]\right),\ldots ,\left(d_{n},c_{n}\left[i\right]\right)$, where i∈{1,2}. To compare the predictive power of entropy, density and Oh’s criterion of SAT/UNSAT, we performed two statistical tests (recall that the data is standardized, and hence comparable):
  – The Δ test: A linear regression test over the series $(e_{1},c_{1}\left[1\right]-c_{1}\left[2\right])\ldots$ $\left(e_{n},c_{n}\left[1\right]-c_{n}\left[2\right]\right)$, and the series $\left(d_{1},c_{1}\left[1\right]-c_{1}\left[2\right]\right)\ldots \left(d_{n},c_{n}\left[1\right]-c_{n}\left[2\right]\right)$.
  – The $\Delta_{{\hat{\beta }}_{1}}$ test: A linear regression test over the series $\left(e_{1},c_{1}\left[1\right]\right)\ldots \left(e_{n},c_{n}\left[1\right]\right)$ and $\left(e_{1},c_{1}\left[2\right]\right)\ldots \left(e_{n},c_{n}\left[2\right]\right)$, and similarly for density (i.e., four tests all together). We then checked the significance of ${\hat{\beta }}_{1}$ for each of these 4 tests. In addition, we checked the hypothesis $H_{0}:{\hat{\beta }}_{1}\left[1\right]-{\hat{\beta }}_{1}\left[2\right]=0$ for each of the measures. The result of this last test appears in the Appendix A.
  Intuitively, the two models tell us slightly different things: the first tells us whether the gap between the two heuristics is correlated with the measure, and the second tells us whether there is a significant difference in the value of ${\hat{\beta }}_{1}$ (the slope of the linear model) between the two heuristics. As we will see in the results, the *p*-value obtained by these models can be very different.
- **Plots**: The plots are based on the original (non-standardized) data. To reduce the clutter (from 5000 points), we rounded all values to 2 decimal points and then *aggregated* them. Aggregation means that points $\left(x,y_{1}\right)\ldots \left(x,y_{n}\right)$ (i.e., *n* points with an equal *x* value) are replaced with a single point (x, $avg(y_{1}\ldots y_{n})$). However the trend-lines in the various plots are depicted according to the *original* data, before rounding and aggregation. The statistical significance of these trend-lines appears in the Appendix A.

## 4. Empirical Findings

### 4.1. The Benchmark Set

All the results that we report below are based on experiments with 10,000 CNF formulas, divided to two equally sized subsets described below. For each such formula, we calculated the exact entropy and solution density, using a combination of the SAT-based model-counters Cachet [4] and SharpSAT [5]. To the best of our knowledge currently these are the most powerful exact model-counters. We note that in addition to SAT-based model-counters, there is also an option of building an Ordered Binary Decision Diagram (OBDD) from the input formula, and then the counting is polynomial in the size of the BDD. The problem is that the BDD itself can become exponential in the number of variables. Some recent examples of applying BDD-based counting include [6,7,8].

The first subset of benchmarks is comprised of 5000 3-SAT random formulas with 100 variables and 400 clauses. These are formulas taken from SAT-lib, in which the number of backbone variables is known. Specifically, there is an equal number of formulas in this set with 10, 30, 50, 70 and 90 backbone variables (i.e., a 1000 formulas of each number of backbone variables), which gave us a near-uniform distribution of entropy among the formulas.

The second subset of benchmarks is comprised of 3296 CNF formulas that are generated with the *modularity*-based CNF generator modularityGen [9], which generates random formulas that have characteristics of real-world problems (out of the 5000 formulas that we generated, this is the number of formulas for which we were able to compute entropy and solution density within the timeout that we set). To understand the structure of these formulas, note first that a CNF formula can be represented by the *variable incidence graph*, which is an undirected graph in which variables are nodes and two nodes are connected if they share a clause. Such a graph can be partitioned into sets of nodes, and each partition is called a *community*. Given a partition we can measure the ratio between the number of edges within communities (in contrast to edges *between* communities) and the number of such edges that we would have gotten had we distributed the same number of edges between the same nodes but in a uniformly random fashion. Between all possible partitions, we take the one that drives this ratio to maximum: this is called the *modularity* of the graph, and is typically denoted by *Q* [10]. It has been recognised in [11] and later in [12] that industrial CNF formulas have high values of *Q* (a “good community structure”), and moreover that heuristics used by modern SAT solvers (unknowingly) exploit this fact for faster solving. In an impressive series of experiments reported in [9], they generated hundreds of formulas with a varying value of *Q*. They then took two solvers, March [13] and Glucose [14], which specialize in random and industrial formulas, respectively, and ran them with those benchmarks as input. Exactly as expected, March was far better with formulas having low values of *Q* and Glucose was much better with formulas having high values of *Q*.

modularityGen gives the user the ability to control, among other things, the modularity *Q*, the number of neighborhoods *n*, and the size of the formula. For our experiments we chose Q=0.7 (based on measurements made in [9], that showed that most industrial formulas have at least this value of modularity, whereas random formulas have values smaller than Q=0.3), n=5, 400 variables and 1600 clauses.

To distinguish between the random and modularity-based benchmarks sets, we will call them respectively *B-Rand* and *B-Mod* from hereon.

### 4.2. Entropy and Density Predict Hardness

**Figure 2:**
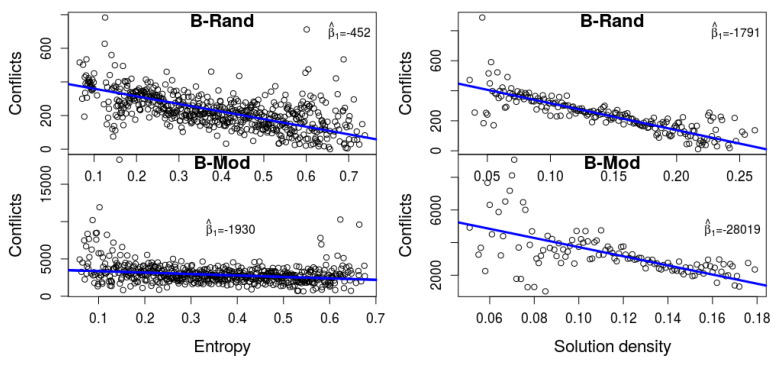
Entropy (**left**) and density (**right**) as predictors of the number of conflicts (based on MiniSat-HACK-999ED). It is apparent that higher entropy and higher density imply a smaller number of conflicts.

We checked the correlation between hardness, as measured by the number of conflicts, and the two measures described above, namely entropy and density. We use the number of conflicts as a proxy of the run-time, because these are all easy formulas for SAT, and hence the differences in run-time are rather meaningless. The two plots in Figure 2 depict this data based on our experiments with the solver MiniSat-HACK-999ED. It is apparent that higher entropy and higher density imply a smaller number of conflicts. It turns out that this is not a characteristic that is unique to this solver. We performed a detailed statistical analysis of seven different solvers, and witnessed a similar phenomenon, as we now describe.

Denote by ${{\hat{\beta }}_{1}}^{E}$ and ${{\hat{\beta }}_{1}}^{S}$ the ${\hat{\beta }}_{1}$-value of the linear models for entropy vs. conflicts and density vs. conflicts, respectively. Table 1 shows strong correlation between both measures to the number of conflicts (the *p*-value in both cases, for all engines, is practically 0). The last two columns show the gap ${{\hat{\beta }}_{1}}^{E}-{{\hat{\beta }}_{1}}^{S}$ and the corresponding *p*-value for $H_{0}:{{\hat{\beta }}_{1}}^{E}-{{\hat{\beta }}_{1}}^{S}=0,H_{1}:{{\hat{\beta }}_{1}}^{E}-{{\hat{\beta }}_{1}}^{S}\neq 0$, when measured across the k=1000 iterations of the bootstrap method that was described in Section 3. For engines with high *p*-value we cannot reject $H_{0}$ with confidence.

### 4.3. A Refinement of Oh’s Results

In this section we describe each of the experiments of Oh [1], and our own version of the experiment based on entropy and density, when applied to the 10,000 benchmarks mentioned above. We omit the details of one experiment, in which Oh examined the effect of canceling database reduction, the reason being that this heuristic is only activated after 2000 conflicts, and most of our benchmarks are solved before that point (we note that our attempt to use an approximate model-counter with larger formulas failed: the inaccuracies were large enough to make the analysis show results that are senseless). Raw data as well as charts and regression analysis of our full set of experiments can be found online in [15].

**Figure 3:**
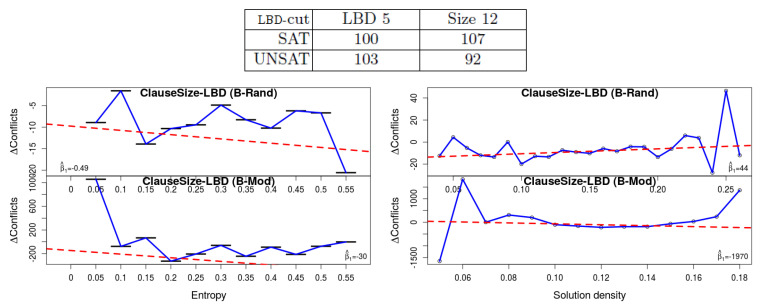
The effect of the deletion criterion. The results of [1] appear in the table at the top, where the numbers indicate the number of solved instances. It shows that for SAT instances keeping everything with clause size 12 is better than keeping everything with LBD 5, whereas the result is opposite for the UNSAT instances. Our own results (**bottom**) are separated by measure (entropy/density) and by the benchmark set. The y-axis corresponds to the difference in the # of conflicts, and hence a positive value shows that the clause-based deletion creates more conflicts. Hence a declining regression line, which is the case in the two entropy experiments (**left**) and in the B-Mod density experiments, shows that with a higher value of entropy (or density), clause-based deletion produces less conflicts in comparison to LBD-based. Hence we see that the effectiveness of the deletion strategy is similar in satisfiable formulas with small entropy and unsatisfiable formulas.

**1. Deletion strategy**: Different solvers use different criteria for selecting the learned clauses for deletion. It was shown in [1] that for SAT instances learned clauses with low Literal Block Distance (LBD) [14] value can help, whereas others have no apparent effect. In one of the experiments, whose results are copied here at the top part of Figure 3, Oh compared the criterion of “core *LBD-cut*” 5 and clause size 12 (an LBD-cut is the lowest value of LBD a learned clause had so far, assuming this value is recalculated periodically). In other words, either save (i.e., do not delete) clauses with an LBD-cut of 5 and lower, or clauses with size 12 or lower. It shows that for UNSAT instances the former is better, whereas the opposite conclusion is reached for the SAT instances. The results of our own experiments are depicted at the bottom of the figure. They show that the latter is indeed slightly better with our benchmarks (all satisfiable, recall). However, what is more important, is that the difference becomes smaller with lower entropy—hence the decline of the trend-lines and the negative $\beta_{1}$ values (recall that the trend-lines are based on the raw data, whereas the diagram itself is computed after rounding and aggregation to improve visibility. Hence occasionally the two do not seem to be perfectly matching). Hence it is evident that formulas with small entropy “behave” more similar to unsat formulas. More information appears in the caption of Figure 3, and in the Appendix A.

**Figure 4:**
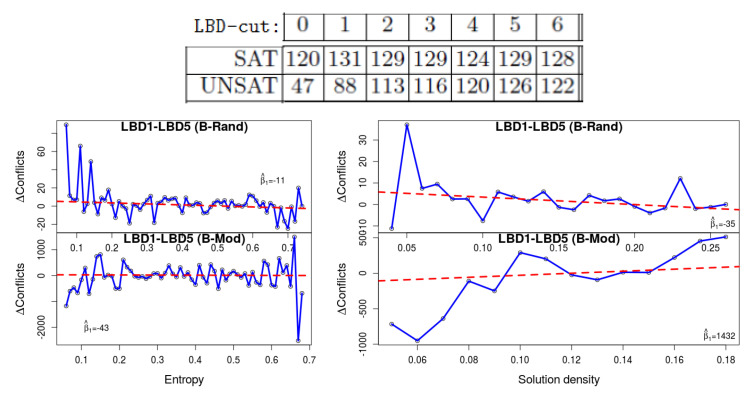
The results of [1] (**top**) show that unsat formulas are solved faster with high LBD-cut. Our results (**bottom**) show that low-entropy and low-density formulas behave more similarly to unsat formulas.

**2. Deletion with different LBD-cut value** Related to the previous heuristic, in [1] it was found that deletion based on larger LBD-cut values, up to a point, improve the performance of the solver with unsat formulas, but not with SAT ones. Figure 4 (top) is an excerpt from his results for various LBD-cut values. We repeated his experiment with LBD-cut 1 and LBD-cut 5. The plots show that lower values of entropy yield a bigger advantage to LBD-cut 5, which again demonstrates that satisfiable formulas with these values ‘behave’ similarly to unsat formulas. The results for density are inconsistent, in the sense that we see the same phenomenon with B-Rand but not with B-Mod.

**3. Restarts policy**: The Luby restart strategy [16] is based on a fixed sequence of time intervals, whereas the Glucose restarts are more rapid and dynamic. It initiates a restart when the solver identifies that learned clauses have higher LBD than average. According to the competitions’ results this is generally better in unsat instances. Oh confirmed the hypothesis that this is related to the restart strategy: indeed his results show that for satisfiable instances Luby restart is better.

**Figure 5:**
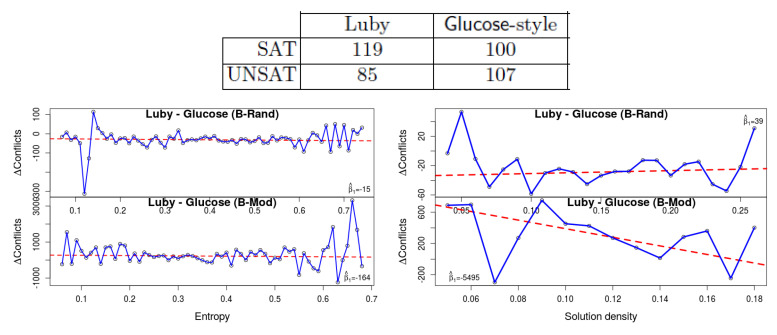
The effect of the restart strategy, comparing Luby and Glucose-style restarts. The results of [1] (**top**) show that the Glucose strategy (rapid restarts) has an advantage in unsat formulas. Our results (**bottom**) show that the same phenomenon is apparent in formulas with low entropy. Indeed observe the decline in the gap: with lower-entropy formulas the number of conflicts is smaller with the Glucose restart strategy.

Our own results can be seen in Figure 5. The fact that the gap in the number of conflicts between Luby and Glucose-style restarts is negative, implies that the former is generally better, which is consistent with Oh’s results for satisfiable formulas. Observe that the trend-line declines with entropy (${\hat{\beta }}_{1}=-15$ and ${\hat{\beta }}_{1}=-164$ for the two benchmark sets), which implies that Glucose restarts are statistically better with low entropy. So again we observe that low entropy formulas ‘behave’ more similar to UNSAT formulas than those that have high entropy. We speculate that with high-entropy instances, the solver hits more branches that can be extended to a solution, hence Glucose’s rapid restarts can be detrimental. Density again seems to have an inconsistent effect between the two benchmark sets.

**Figure 6:**
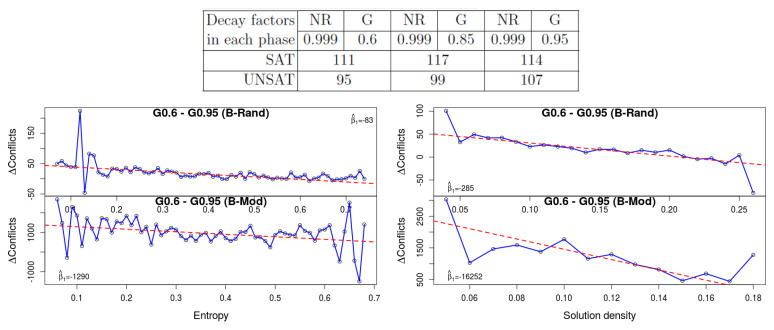
The effect of variable decay: the results of [1] (**top**) generally show that unsat formulas are better solved with a high *decay factor*. The restart policy in his solver is hybrid: it alternates between a “no-restart” (NR) phase and a “Glucose” (G) phase. The “NR” and “G” columns hold the decay factor during these phases. The plots at the bottom show the gap in the number of conflicts between G=0.6 and G=0.95. It shows that with low entropy, strong decay (i.e., G=0.6) is worse, similar to the effect that it has on unsat formulas. With low density (**right**) a similar effect is visible.

**4. The variable decay factor**: This experiment shows the most consistent and the most statistically significant results. The well-known VSIDS branching heuristic is based on an activity score of literals, which decay over time, hence giving higher priority to literals that appear in recently learned clauses. In the solver MiniSat_HACK_999ED, there is a different decay factor for each of the two *restart phases*: this solver alternates between a Glucose-style (G) restart policy phase and a no-restart (NR) phase (these two phases correspond to good heuristics for SAT and UNSAT formulas, respectively). In [1] Oh compares different decay factors for each of these restart phases on top of MiniSat_HACK_999ED. His results show that for UNSAT instances slower decay gives better performance, while for SAT instances it is unclear. His results appear at the top of Figure 6. We experimented with the two extreme decay factors in that table: 0.95 and 0.6. Please note that since our benchmarks are relatively easy, the solver never reaches the NR phase. The plot shows the gap in the number of conflicts between these two values. A higher value means that with strong decay (0.6) the results are worse. We can see that the results are worse with strong decay when the entropy is low, which demonstrates again that the effect of the variable decay factor is similar for unsat formulas and satisfiable formulas with low entropy. A similar phenomenon happens with small density in both benchmark sets.

## 5. Conclusions

We defined the *entropy* property of satisfiable formulas, and used it, together with solution density, to further refine and investigate the results achieved by Oh in [1]. We began by showing a clear correlation between these two measures and the number of conflicts in Section 4.2, based on an analysis of seven different solvers. We then showed that both measures predict better the effect of various SAT heuristics than Oh’s sat/unsat divide, and that satisfiable formulas with small entropy ‘behave’ similarly to unsatisfiable formulas. In that respect the entropy measure is more consistent than density across benchmarks types. Since both measures are hard to compute we do not expect these results to be applied directly (e.g., in a portfolio), but perhaps future research will find ways to cheaply approximate them and lead to improved heuristics.

## Figures and Tables

**Table 1:** For each solver, we list the 95% confidence interval of its ${\hat{\beta }}_{1}^{E}$ (entropy) and ${\hat{\beta }}_{1}^{S}$ (solutions). For all engines the corresponding *p*-value is practically 0 (i.e., ≤$10^{-100}$). The last two columns refer to the gap between these measures.

Solver	${\hat{\beta }}_{1}^{E}$	${\hat{\beta }}_{1}^{S}$	${\hat{\beta }}_{1}^{E}-{\hat{\beta }}_{1}^{S}$	*p*-Value
MiniSat-HACK-999ED	(−84.29, −72.58)	(−84.93, −73.56)	(5.37, 16.96)	0.716
MiniSat-HACK-999ED	(−86.31, −75.36)	(−82.97, −72.64)	(−7.51, 1.44)	0.200
(modified to luby)				
MiniSat-HACK-999ED	(−72.84, −63.61)	(−72.31, −62.91)	(−4.80, 3.57)	0.738
(modified for 2 phases)				
SWDiA5BY	(−91.61, −79.17)	(−90.97, −78.77)	(−5.95, 4.92)	0.84
COMiniSatPS	(−74.68, −64.58)	(−75.41, −65.43)	(−3.79, 5.37)	0.76
lingeling-ayv	(−76.19, −66.61)	(−71.70, −61.76)	(−8.99, −0.35)	0.029
Glucose	(−91.24, −79.34)	(−90.56, −78.88)	(−6.00, 4.85)	0.845

## Appendix A. Regression-Tests Results

Table A1 lists the confidence interval and corresponding *p*-value, for the two regression tests Δ and $\Delta_{{\hat{\beta }}_{1}}$ (in the latter we also list the results for ${\hat{\beta }}_{0}$) that were explained in Section 3, and the four experiments described in Section 4. $H_{1}$ is one-sided.

**Table A1:** Regression-tests results for the four experiments in Section 4. *p*-value $\leq 10^{-10}$ are rounded to 0.

Exp.	Bench.	Measure	Confidence	*p*-Val	Confidence	*p*-Val	Confidence	*p*-Val
			Interval (Δ)		interval ($\Delta_{{\hat{\beta }}_{1}}$)		Interval ($\Delta_{{\hat{\beta }}_{0}}$)	
1	B-Rand	Entropy	(−17.207, 16.04)	0.48	(−17.878, 16.155)	0.05	(−15.836, −2.328)	0
	B-Mod	Entropy	(−806.83, 675.0)	0.46	(−779.3, 699.96)	0.44	(−387.3, 164.9)	0.003
	B-Rand	Density	(−21.39, 108.65)	0.09	(−19.30, 111.97)	0.39	(−25.01, −5.53)	0
	B-Mod	Density	(−6829, 2647)	0.2	(−6663, 2883)	0.16	(−519.4, 758.4)	0.003
2	B-Rand	Entropy	(−24.16, 2.49)	0.04	(−24.59, 1.71)	0.39	(0.640, 11.138)	0.01
	B-Mod	Entropy	(−651.6, 550.48)	0.44	(−622.03, 545.26)	0.49	(−190.64, 251.56)	0.33
	B-Rand	Density	(−86.37, 17.50)	0.09	(−85.24, 16.11)	0.47	(−0.723, 14.450)	0.01
	B-Mod	Density	(−2472, 5240)	0.23	(−2578, 5455)	0.13	(−717.8, 371.4)	0.33
3	B-Rand	Entropy	(−53.50, 22.17)	0.22	(−52.71, 23.33)	0.001	(−40.55, −10.27)	0
	B-Mod	Entropy	(−938.9, 570.7)	0.33	(−921.4, 611.0)	0.48	(−4.2, 553.3)	2 × 10${}^{-6}$
	B-Rand	Density	(−101.57, 183.8)	0.30	(−105.89, 183.08)	0.05	(−55.67, −13.35)	0
	B-Mod	Density	(−9939, −937)	0.007	(−9991, −832)	0.16	(308.4, 1543.6)	2 × 10${}^{-6}$
4	B-Rand	Entropy	(−96.55, −68.38)	0	(−97.27, −69.05)	0.125	(42.11, 53.69)	0
	B-Mod	Entropy	(−2042, −559)	0.0004	(−2091, −529)	0.38	(1127, 1717)	0
	B-Rand	Density	(−340.3, −224.1)	0	(−343.9, −226.5)	0.47	(50.44, 67.76)	0
	B-Mod	Density	(−21,540, −11,007)	0.003	(−21,474, −10,950)	0.46	(2357, 3788)	0
